# Circulating PD1^+^Vδ1^+^γδ T Cell Predicts Fertility in Endometrial Polyp Patients of Reproductive-Age

**DOI:** 10.3389/fimmu.2021.639221

**Published:** 2021-06-15

**Authors:** Xiao-Hong Li, Mei-Yin Lu, Yi-jia Li, Zong-hua Liu, Zhi-nan Yin, Bin Liu, Yang-zhe Wu

**Affiliations:** ^1^ Department of Obstetrics and Gynecology, Shenzhen Baoan Women’s and Children’s Hospital, Jinan University, Shenzhen, China; ^2^ Department of Biobank, Shenzhen Baoan Women’s and Children’s Hospital, Jinan University, Shenzhen, China; ^3^ Zhuhai Institute of Translational Medicine, Zhuhai People’s Hospital (Zhuhai Hospital Affiliated with Jinan University), Zhuhai, China; ^4^ The Biomedical Translational Research Institute, Faculty of Medical Science, Jinan University, Guangzhou, China

**Keywords:** endometrial polyp, fertility, biomarker, γδ T cells, PD-1

## Abstract

Clinically, immune cell function is correlated with pathogenesis of endometrial polyp (EP) and infertility of women of reproductive-age. However, the underlying immune cell hallmark in EP patients remains unclear. Here, we focused on analyzing circulating immune cells, and attempted to reveal the correlation between peripheral immune cell functional phenotypes and fertility in EP patients. Through comparison of circulating CD4^+^/CD8^+^ T cells, NK cells, and γδ T cells between 64 EP patients and 68 healthy females, we found that γδ T cells, but not CD4^+^/CD8^+^ T cells and NK cells, were immunologically correlated with conception rate and conception interval time. Specifically, total γδ T cells and the Vδ1^+^PD1^+^ γδ T subpopulation decreased whereas the Vδ1/Vδ2 ratio increased in EP patients compared to healthy controls. Moreover, the patients with the higher Vδ1/Vδ2 ratio (median value equals 1.04) had a poorer fertility and longer interval time of conception (210 days *versus* 158 days for control). Meanwhile, higher Vδ1^+^PD1^+^ γδ T cell proportion (median equals 15.7) was positively correlative with both higher conception rate and shortened median conception interval time (130 days for Vδ1^+^PD1^high^ group *versus* 194 days for Vδ1^+^PD1^low^ group). Notably, in healthy controls, both Vδ1/Vδ2 ratio and Vδ1^+^PD1^+^ γδ T cell proportion correlated with pregnancy rate oppositely, comparing to EP patients. Together, our results suggested that imbalanced γδ T cell population occurred in EP patients, and that Vδ1/Vδ2 ratio and PD-1 expression of Vδ1^+^ γδ T cells could be potentially developed into valuable predictors for fertility in EP patients.

## Introduction

The dynamic equilibrium is one type of fundamental features of human endometrium, for example, rebalancing between periodical proliferation and apoptosis, tissue breakdown, and repairment, which serves the major and basic function of regulating embryo implantation (1). Therefore, the excessive proliferation of endometrium would occur in certain condition of inductions, and this could adversely induce diseases if it keeps getting worse. Endometrial polyp (EP) is one consequence of excessive proliferation of endometrium commonly occurring in women of reproductive-age. Clinically, EP is a type of benign lesion in women with focal growth of the endometrial glands and stroma (2). EP affects approximately 7.8–34.9% of reproductive women and usually impairs fertility (3, 4). Previous reports showed that assisted reproductive technology such as *in vitro* fertilization is required for 11–45% EP patients (5, 6). In addition to fertility impairment, abnormal uterine bleeding (AUB) is another common symptom of EP, which relates to aberrant tissue damage and repair in endometrium. Currently, it generally recognized that EP can negatively affect the quality of life, such as physical, emotional, sexual, and professional aspects of the lives of women (7).

As for EP diagnosis, transvaginal ultrasonography (TVUS) is a reliable approach, and the Color-flow or Power Doppler technology can further improve the diagnostic accuracy of TVUS (1). Blind dilation and curettage or biopsy is only used to exclude the malignant polyps (1). For small or asymptomatic polyps, conservative management rather than medical treatments (GnRHa or hormonal therapy) is generally adopted clinically (2). For AUB or infertility patients, however, surgeries including blind dilation and curettage, hysteroscopic polypectomy, and even hysterectomy could be applied to remove polyps (1). Published clinical data showed that polypectomy could effectively relieve symptoms of EP (3, 4). Nevertheless, infertility is a common issue in EP patients of reproductive-age (5). Currently, the underlying mechanism of EP occurrence or development remains to be fully illustrated. Moreover, the biomarkers associating with fertility in EP patients are urgently needed clinically, which could particularly help the EP patient who is planning pregnancy.

Although the precise pathogenesis of EP remains largely unclear, high risk factors such as aging, hypertension, obesity, and drug (e.g. tamoxifen) usage were reported to contribute to development of EP (1). Increasingly, immunological imbalance or dysfunction has been recognized as one of important factors causing EP (6–8). Black et al. (2013) reported that inflammatory lesion associated with infiltrated mast cells in EP tissue plays important roles in inducing local immune disturbances (8). Moreover, the circulating monocytes of EP patients can secrete high levels of TNF, IL-1β, IL-6, and IL-23, which could impair fertility (9). In addition, endometrial inflammation (10, 11) or infection of chlamydia trachomatis (12) are also prone to induce AUB and subfertility (10). Altogether, local inflammation and dysfunctional cellular immune environment are believed to play important roles in etiology, pathophysiology, and infertility or poor pregnancy outcomes in EP patients.

In the present study, we focused on functional phenotype alterations of circulating immune cells (CD4+/CD8+ T cells, NK, and γδ T cells) in EP patients, and aimed to discover immune cell related biomarkers associating with pregnancy ability. We used our previously established methodology (13) to globally evaluate phenotypes of peripheral immune cells, and compare alterations between healthy populations and EP patients of reproductive age. Then we analyzed the immunological relevance between the immune cell phenotypes and pregnancy outcome, indicating that circulating PD1+Vδ1+γδ T cell and the Vδ1/Vδ2 ratio could be developed into the prediction biomarkers for pregnancy ability in endometrial polyp patients of reproductive-age. This proof-of-concept work not only will benefit further investigation of the underlying pathogenesis of EP, but also could promote development of immune cell phenotype biomarkers predicting pregnancy ability in EP of reproductive age.

## Materials and Methods

### Enrollment and Follow-Up of Research Cohorts

**Figure 1:**
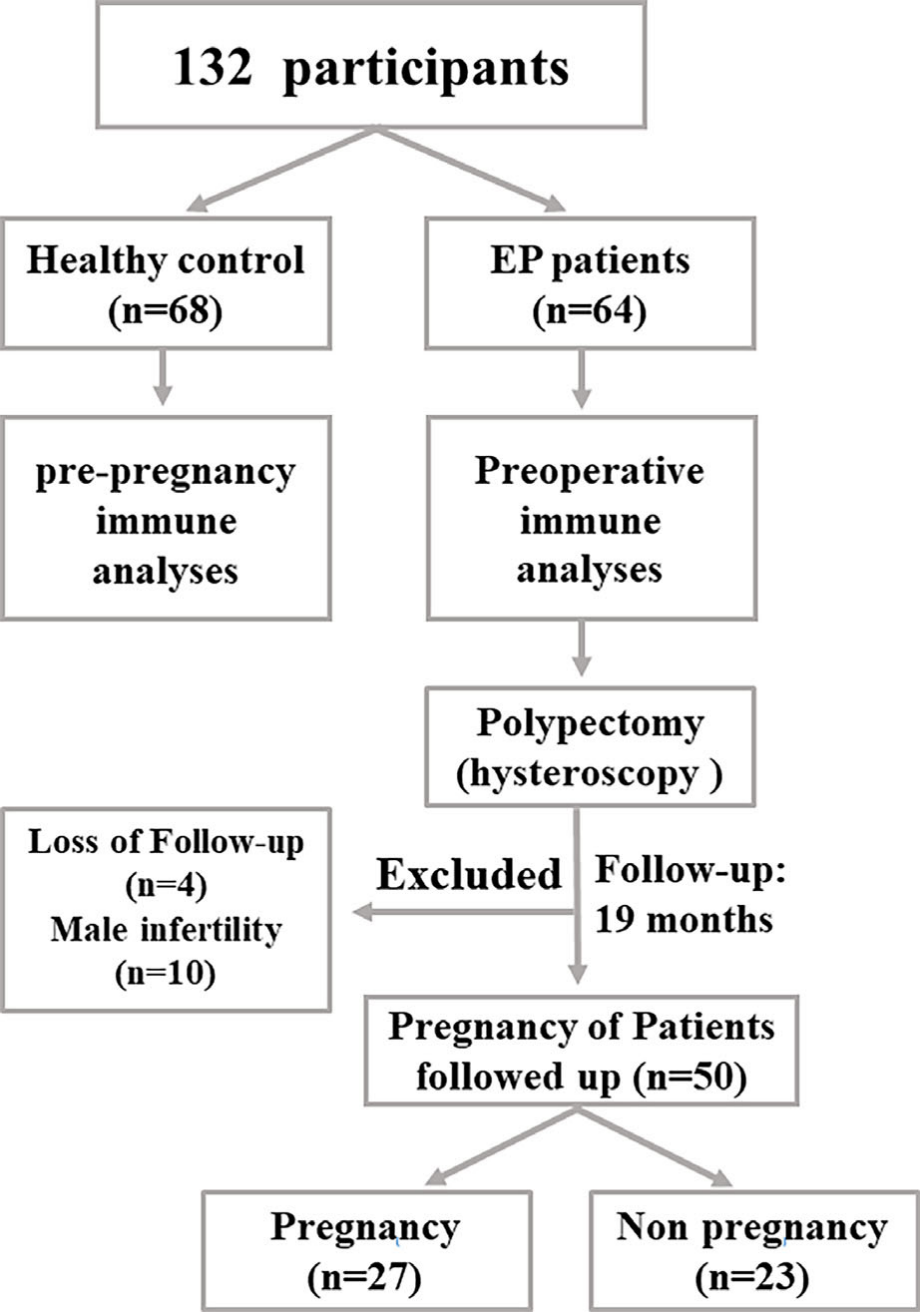
The flow chart showing how this study was designed. In our work, 64 EP patients and 68 healthy population (control group) were enrolled to collect samples and perform subsequent analyses.

The research cohort of this study was shown in **Figure 1**. Briefly, 64 EP patients with 20~40 years old routinely diagnosed by hysteroscopy (1), ultrasonic imaging, and pathological examinations were enrolled at Shenzhen Baoan Women’s and Children’s Hospital of Jinan University between June and December 2018. Clinically, the common treatment of EP patients is to apply hysteroscopic polypectomy. Therefore, to evaluate peripheral immune cell phenotypes, 2 ml of heparin anticoagulant blood was collected before operation of polypectomy. At the same time, 68 healthy females of reproductive age who were going to receive pre-pregnancy medical examinations were enrolled in this study as well. The excluding criteria of the healthy cohort included endometrial polyps, ovary morbidities, infertility, leiomyoma, and other tumors. Similarly, 2 ml of peripheral blood was collected for subsequent analyses.

As for follow-up, all enrolled people were scheduled to come back for routine examinations every 3 months. The pregnancy status was verified by serum human chorionic gonadotropin (HCG) and ultrasound, and the first day of the last menstrual period (LMP) was recorded. The pregnancy status of two cohorts was followed up until 2019 December. Then the association between peripheral immune cell functional phenotypes and pregnancy rate was analyzed.

This study was approved by the Ethics Committee of Shenzhen Baoan Women’s and Children’s Hospital, Jinan University (IRB No: LLSC-2018-08-01).

### Immune Cell Phenotype Analyzing by Flow Cytometry

Collected peripheral blood samples were analyzed using flow cytometry. Briefly, peripheral blood mononuclear cells (PBMCs) were isolated using Ficoll-Paque centrifugation, then stained by the following antibodies: PerCP-CY5.5-conjugated anti-CD3; APC-conjugated anti-CD4; FITC-conjugated anti-CD45RA; PE-conjugated anti-CD25 and anti-Vδ2; PE-CY7-conjugated anti-CD28 and anti-NKG2D; BV421-conjugated anti-56, anti-127, anti-CD194, and anti-TCRγδ; BV510-conjugated anti-CD8 and anti-NKP46; Alexa Fluor 647-conjugated anti-CCR7 and anti-CXCR5; Alexa Fluor 484-conjugated anti-CD183, and BB515-conjugated anti-PD-1. The antibodies were bought from BD biosciences. Samples were then analyzed using BD LSR Fortessa (BD Biosciences) at Shuangzhi Purui Medical Laboratory Co., Ltd. (China), and data was analyzed using FlowJo 10.1 software (Tree Star Inc., Ashland, OR, USA).

### Statistical Analysis

SPSS 25.0 (IBM) was used to process and analyze the data. For analyzing the difference in immune cells and its subsets between patients and controls, Mann-Whitney U tests were performed. The pregnancy rate and median pregnancy time (MPT) between different groups were analyzed by Kaplan-Meier Survival Curve, log-rank test, and Cox regression analysis by adjusting for age, history of infertility, and the number and diameter of endometrial polyp in EP patients. All tests were two sided, and the level of significance was set at 0.05. Statistical figures were produced by GraphPad Prism7.0 (GraphPad Software, USA).

## Results

### Overall Immune Function Profiles of γδ T, αβ, and NK Cells in EP Patients

**Figure 2:**
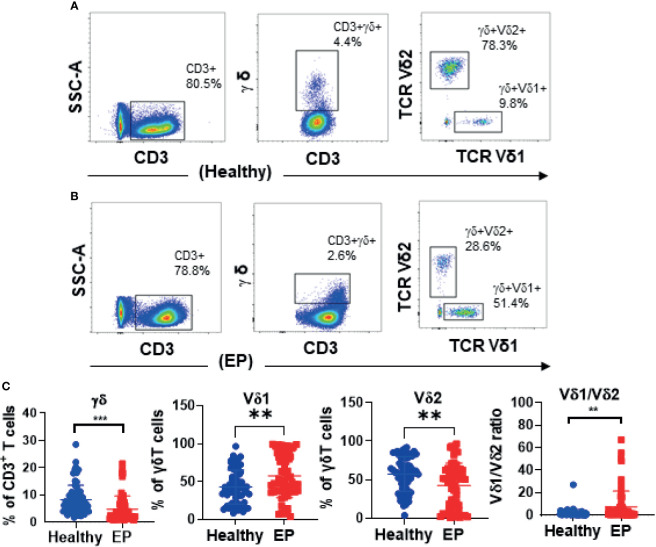
Representative flow graphs **(A, B)** and statistical analysis results **(C)**. **(C)** Statistical comparison of γδ T cell proportions in CD3+ T cells, Vδ1 and Vδ2 subsets in γδ T cells, and the Vδ1^+^/Vδ2^+^ ratio between the healthy group and the patient group. **P < 0.01. ***P < 0.001.

**Figure 3:**
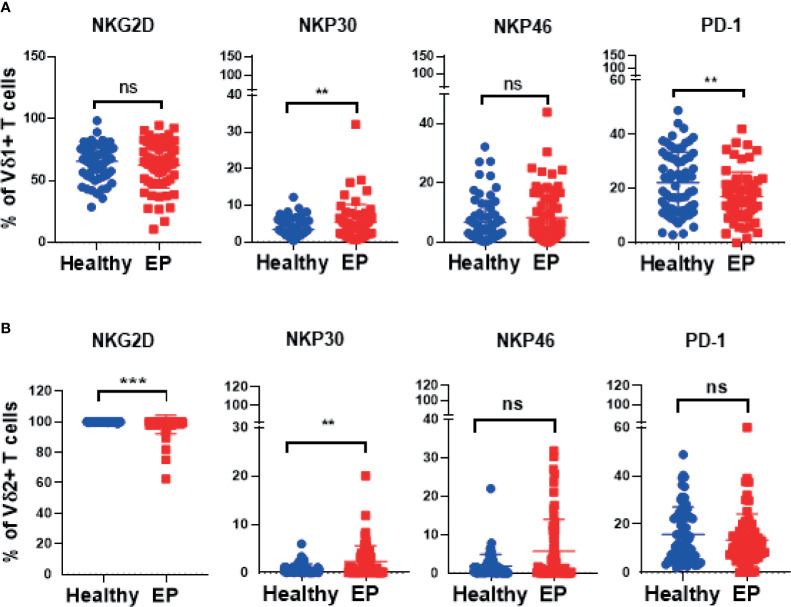
Expressions of crucial receptors of Vδ1^+^ and Vδ2^+^ γδ T cells in healthy and EP populations. **(A)** Comparison analyses of expressions of NKG2D, PD-1, NKP30, and NKP46 receptors of Vδ1 γδ T cells acquired by flow cytometry. **(B)** Expressions of NKG2D, PD-1, NKP30, and NKP46 receptors expressed in Vδ2 cell subset. ns, no significance; **P < 0.01; ***P < 0.001.

In our work, 64 EP patients were enrolled, and 68 healthy women were included as control group, and the cohort diagram is shown in **Figure 1**. The clinical and demographic characteristics of these EP patients and healthy controls were described as **Table 1**. Given the unique immune function of γδ T cells, it’s of significant importance to detect how γδ T cells were proportionally altered in patients. We thus compared cell proportion of γδ T cells (**Figures 2** and **3**) between control and EP groups using flow cytometry. It showed that, compared with healthy populations, EP patients have statistically lower amount of γδ T cells (**Figure 2B**). We further analyzed statistical alterations of γδ T subsets. It showed that EP has higher Vδ1 cell proportion (57.47± 3.62, n = 64) comparing with the control population (42.72± 2.71, n = 68), but the lower Vδ2 cell proportion (42.32± 3.63) comparing with the control (57.2± 2.72). This thus led to a significant elevation of the Vδ1/Vδ2 ratio, from 1.49± 0.41 for the control to 7.16± 1.77 for the EP patients (**Figure 2C**). Further flow analyses on Vδ1^+^γδ T cells indicated that Vδ1^+^NKP30^+^ γδ T cells increased while Vδ1^+^PD-1^+^ γδ T cells decreased in patients (**Figure 3A**), and representative flow gating graphs are shown in **Supplementary Figure 1**. As for Vδ2^+^γδ T cells, we observed that Vδ2^+^NKG2D^+^ γδ T cells decreased while Vδ2^+^NKP30^+^ γδ T cells increased in EP patients (**Figure 3B**).

**Table 1:** Clinical and demographic characteristics of endometrial polyp patients and healthy controls.

Variables	Cases, n (%)	Controls, n (%)	*P* ^a^
All subjects	64 (100)	68 (100)	
Age, years			
<30	27 (42.2)	36 (52.9)	0.288
≥30	37 (57.8)	32 (47.1)	
Menstrual cycle, days			0.384
<24	0 (0)	2 (2.9)	
24–35	56 (87.5)	58 (85.3)	
>35	8 (12.5)	8 (11.8)	
Menstrual duration, days			0.190
≤7	54 (84.4)	50 (73.5)	
>7	10 (15.6)	18 (26.5)	
Dysmenorrhea	53 (82.8)	37 (60.3)	<0.001
History of infertility	18 (28.1)	0 (0.0)	<0.001
AUB	14 (21.9)	0 (0.0)	<0.001
Gravidities			0.832
0	29 (45.3)	28 (41.2)	
1	16 (25.0)	20 (29.4)	
≥2	19 (29.7)	20 (29.4)	
Abortion			0.122
0	47 (73.4)	39 (57.4)	
1	10 (15.6)	20 (29.4)	
≥2	7 (11.0)	9 (13.2)	
Deliveries			0.005
0	34 (53.1)	39 (60.9)	
1	21 (32.8)	29 (45.3)	
≥2	9 (14.1)	0 (0)	
Red cells (×10^12^/L)			0.987
<4.42	30 (46.9)	33 (48.5)	
≥4.42	34 (53.1)	35 (51.5)	
Hemoglobin (g/L)			0.001
<134	50 (78.1)	32 (47.1)	
≥134	14 (21.9)	36 (52.9)	
WBC (×10^9^/L)			0.707
<5.86	28 (43.7)	33 (48.5)	
≥5.86	36 (56.3)	35 (51.5)	
Lymphocytes (×10^9^/L)			0.569
<2.09	38 (59.4)	36 (52.9)	
≥2.09	26 (40.6)	32 (47.1)	
FSH (IU/L)			
<8.42	27 (42.2)	–	
≥8.42	37 (57.8)	–	
LH (IU/L)			
<4.50	22 (34.4)	–	
≥4.50	42 (65.6)	–	
Testosterone (nmol/L)			
<1.51	21 (32.8)	–	
≥1.51	43 (67.2)	–	
E2 (pmol/L)			
<187	26 (40.6)	–	
≥187	38 (59.4)	–	
Progesterone (nmol/L)			
<3.17	33 (51.6)	–	
≥3.17	31 (48.4)	–	
Prolactin (ug/L)			
<12.57	21 (32.8)	–	
≥12.57	43 (67.2)	–	
Endometrial thickness, cm	0.8 ± 0.3		
<0.8	26 (40.6)	–	
≥0.8	38 (59.4)	–	
Multiple polyps	43 (67.2)	–	
Diameter of polyp, cm	1.1 ± 0.5	–	
>1	26 (40.6)	–	
≤1	38 (59.4)	–	
Pregnancy time after follow-up (days)	185 ± 119	142 ± 131	0.074
Pregnancy after follow-up	27 (42.2)	35 (51.5)	0.372

a Chi-square test.

AUB, abnormal uterine bleeding; WBC, white blood cells; FSH, follicle-forming hormone; E2, estradiol; LH, luteinizing hormone.

Here, we also compared changes of αβ T and NK cells between control and EP groups (**Supplementary Figures 2, 3**) by checking expressions of function-related surface marker receptors. Here, CCR7 and CD45RA were used to determine CD4^+^ and CD8^+^ T cell differentiation, it indicated that naïve (CD4+CCR7+CD45RA+) and central memory (CM, CD4+CCR7+CD45RA−) populations statistically increased in EP patients, and terminal differentiated effector memory (EMRA, CD4+CCR7−CD45RA+) populations statistically decreased in patients (**Supplementary Figure 2A**). As for CD8^+^ T cells, we found that CM subset significantly increased while EMRA subset decreased in patients compared with control group (**Supplementary Figure 2B**).

Given helper T (Th, CD3^+^CD4^+^CXCR5^−^) and follicular helper T (Tfh, CD3^+^CD4^+^CXCR5^+^) cells are all differentiated from the naïve CD4^+^ T cells, we also compared the proportional alterations of these cells between patients and healthy controls. We found that, compared to the control group, the ratios of Th1/Th2, (Th1+Th17)/Th2, and Tfh1/Tfh2 were significantly higher in patients (**Supplementary Figures 2C, D**). Additionally, we also analyzed double negative T cells (CD3^+^CD4^−^CD8^−^) and CD56^+^NKP30^+^ NK cells, it revealed that the double negative T cells were statistically increased in patients compared with the healthy population, and meanwhile CD56^+^NKP30^+^ NK cells were statistically lowered (**Supplementary Figure 3**).

### Vδ1^+^PD1^+^ γδ T Cells Associate With Pregnancy Rate of EP Patients

**Figure 4:**
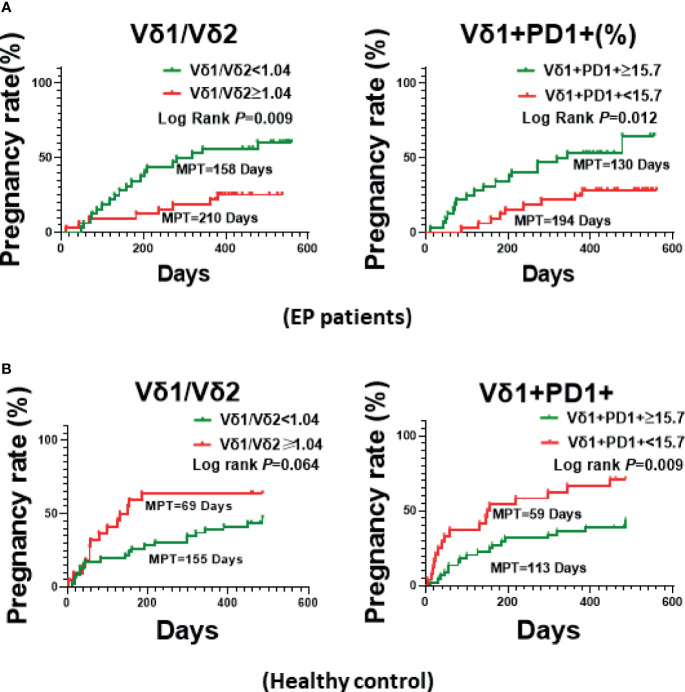
The Kaplan-Meier Curve plotted based on the median values of Vδ1^+^/Vδ2^+^ ratio or Vδ1^+^PD-1^+^ γδ T cells of EP patients. **(A)** Comparison of pregnancy rate (MPT) between two groups with above or below Vδ1/Vδ2 ratio (1.04), or PD-1 expression (15.7%). **(B)** Comparison pregnancy rate (MPT) between healthy populations.

To uncover which immune cell subset was the most important factor associating with pregnancy rate of EP patients, we further analyzed correlation between pregnancy rate and immune cell subsets in 49 EP patients, as shown in **Figure 4** and **Supplementary Figure 3**. We could clearly see that, among 15 immune cell subsets/parameters, only Vδ1^+^PD1^+^ γδ T cell and the Vδ1/Vδ2 ratio were closely relevant with pregnancy rate of EP patients (**Table 2**, **Figure 4A**). Specifically, in peripheral blood of EP patients, Vδ1^+^ subset is reversely correlated with pregnancy rate. Herein, the median value 1.04 could be used as the threshold value of Vδ1/Vδ2 ratio to predict the pregnancy rate of EP patients (adjusted relative risk (RR) = 0.330, 95% CI = 0.137–0.791). It showed that the median pregnancy time (MPT) prolonged from 158 days (median value **<**1.04) up to 210 days (median value ≥1.04). As for the proportion of PD-1^+^ cells in Vδ1^+^γδ T cells, we found that 15.7 (median value) could be used as threshold value of Vδ1^+^PD1^+^ γδ T cell to evaluate the pregnancy rate of EP patients (adjusted RR = 0.293, 95% CI = 0.125–0.685), and we found that 130 days for median value of Vδ1^+^PD1^+^ ratio ≥15.7 and 194 days for **<**15.7. Such results indicated higher Vδ1/Vδ2 ratio and lower Vδ1^+^PD-1^+^ γδ cell levels were associated with a poor post-operative pregnancy rate in EP patients. Then we used markers CCR7 and CD45RA to determine whether CD4+ T cell differentiation were different between patients and healthy population. Results were exhibited in **Supplementary Figure 4B**, showing increases of naïve (CD4+CCR7+CD45RA+), central memory (CM, CD4+CCR7+CD45RA−), and terminal differentiated effector memory (EMRA, CD4+CCR7−CD45RA+) populations in patients (all *P* values **<**0.05).

**Table 2:** Analysis of Vδ1/Vδ2, Vδ1^+^PD1^+^ γδ T cells and pregnancy rate.

	Pregnants *n* (%)	MPT (days)	Log-Rank *p* Value	RR (95% CI)^a^	Cox model *p* Value ^a^
**Patients**					
**Vδ1/Vδ2**	27 (42.2)	170			
<1.04	19 (59.4)	158		1 (ref.)	
≥1.04	8 (25.0)	210	0.009	0.330 (0.137–0.791)	0.013
**Vδ1+PD1+**	27 (42.2)	170			
≥15.7	18 (56.3)	130		1 (ref.)	
<15.7	9 (28.1)	194	0.012	0.293 (0.057–0.662)	0.005
**Controls**					
**Vδ1/Vδ2**	35 (51.5)	100			
<1.04	21 (45.7)	155		1 (ref.)	
≥1.04	14 (63.6)	69	0.064	0.521 (0.263–1.034)	0.062
**Vδ1+PD1+**	35 (51.5)	100			
≥15.7	18 (40.9)	113		1 (ref.)	
<15.7	17 (70.8)	59	0.009	0.399 (0.204–0.780)	0.007

MPT, median pregnancy time; RR, relative risk; ref., reference.

a Cox regression analysis was adjusted for age, history of infertility, and the number and diameter of endometrial polyp in EP patients; adjusted for age and history of infertility in controls.

As for healthy controls (**Figure 4B**, **Table 2**), however, we found that, higher Vδ1/Vδ2 ratio (≥1.04) was linked with better pregnancy rate (69 days of MPT for ≥1.04, but 155 days for <1.04; adjusted RR = 0.521, 95% CI = 0.263–1.034), but higher Vδ1^+^PD1^+^ γδ T cell proportion (≥15.7) associated with attenuated pregnancy ability (113 days of MPT for ≥15.7, but 59 days for <15.7; adjusted RR = 0.399, 95% CI = 0.204–0.780). Additionally, we also checked CD3^+^CD4^−^CD8^−^T cells, CD56^+^NKP30^+^ NK cells, the differentiated subsets of CD4^+^ and CD8^+^ T cells, the ratios of Th1/Th2 and Tfh1/Tfh2, and other parameters of EP patients, it turned out that there was no statistical difference between groups of respective statistical median values (**Supplementary Figure 4**).

## Discussion

Although reproductive immunology has achieved great progresses over past decades (14–16), and the view “immune suppression” during pregnancy has been widely accepted as one of important conceptions in the field of reproductive immunology (15, 16), further insights into immunity features of women of reproductive-age still remains to be fully illuminated. Although previous study showed that various immune cells, including natural killer (NK) cells (17), macrophages (9), and CD4^+^ T cells (18), play key roles in maintaining normal pregnancy at term, however, how peripheral immunity affects pregnancy ability is yet to be fully understood. Therefore, in this prospective study, we focused on characterizing immune profiles/phenotypes of circulating immune cells, such as CD4^+^ T cells, CD8^+^ T cells, NK cells, and γδ T cells. As for CD4^+^ and CD8^+^ T cells, we checked 22 parameters, including CD3^+^, CD3^+^CD4^+^, CD3^+^CD8^+^, CD3^+^CD4^+^CD8^+^, CD3^+^CD4^−^CD8^−^, CD3^+^CD4^+^/CD3^+^CD8^+^, CD3^+^CD4^+^CD45RA^+^CCR7^+^, CD3^+^CD4^+^CD45RA^+^CCR7^−^, CD3^+^CD4^+^CD45RA^−^CCR7^+^, CD3^+^CD4^+^CD45RA^−^CCR7^−^, CD3^+^CD4^+^CD28^−^, CD3^+^CD4^+^CD28^+^, CD3^+^CD4^+^CD25^+^CD127^−^, CD3^+^CD8^+^CCR7^+^CD45RA^+^, CD3^+^CD8^+^CCR7^−^CD45RA^+^, CD3^+^CD8^+^CCR7^+^CD45RA^−^, CD3^+^CD8^+^CCR7^−^CD45RA^−^, CD3^+^CD8^+^CD28^−^, CD3^+^CD8^+^CCR7^−^CD45RA^−^CD127^+^, CD3^+^CD8^+^CCR7^−^CD45RA^+^CD127^+^, CD3^+^CD8^+^CCR7^−^CD45RA^−^CD127^−^, and CD3^+^CD8^+^CCR7^−^CD45RA^+^CD127^−^ T cells. Meanwhile, we checked 10 parameters for NK cells, including CD3^+^CD56^+^ (TNK), CD3^−^CD56^+^, CD3^−^CD56^high^, CD3^−^CD56^low^, (CD3^−^CD56^high^)/(CD3^−^CD56^low^), CD3^−^CD56^+^CD94^+^KIR^−^, CD3^−^CD56^+^CD94^−^KIR^+^, CD3^−^CD56^+^NKG2D^+^, CD3^−^CD56^+^NKP30^+^, and CD3^−^CD56^+^NKP46^+^. Moreover, we further analyzed phenotypes of circulating γδ T cells, the 12 parameters contained CD3^+^γδ^+^, CD3^+^γδ^+^Vδ1^+^, CD3^+^γδ^+^Vδ2^+^, Vδ1^+^/Vδ2^+^ ratio, CD3^+^γδ^+^Vδ2^+^NKG2D^+^, CD3^+^γδ^+^Vδ2^+^PD1^+^, CD3^+^γδ^+^Vδ2^+^NKP30^+^, CD3^+^γδ^+^Vδ2^+^NKP46^+^, CD3^+^γδ^+^Vδ1^+^NKG2D^+^, CD3^+^γδ^+^Vδ1^+^PD1^+^, CD3^+^γδ^+^Vδ1^+^NKP30^+^, and CD3^+^γδ^+^Vδ1^+^NKP46^+^. Among these 44 immune parameters, we only observed 16 parameters had statistical difference (refers to **Figures 2**, **3** and **Supplementary Figures 2, 3**). Such results suggested that only partial immune parameters of peripheral immune cells (CD4^+^, CD8^+^ T cells, NK cells, and γδ T cells) would be relevant to pregnancy of reproductive-age of women in the context of endometrial polyp (EP). Given this context, we further conducted correlation analyses between immune parameters with statistical difference and pregnancy rate, and the results surprisingly showed that only two parameters Vδ1^+^/Vδ2^+^ ratio and CD3^+^γδ^+^Vδ1^+^PD1^+^ T cell proportion, rather than all other parameters, were immunologically correlated with pregnancy rate.

It should be mentioned here that, γδ T cell along with αβ T cell are two major subsets of T lymphocytes. Different from αβ T cell, γδ T cell only constitutes 1–10% of peripheral blood T lymphocytes in human, and majority of cells locates in tissue mucosa (19), such as uterine endometrium (20). Unlike αβ T cells, the activation of γδ T cells is MHC-independent (21). Importantly, increasing evidences suggested that γδ T cells can exert strong direct or indirect influences on network of both innate and adaptive immunity (22), for instance, activation of antigen-presenting cells and/or direct stimulation of other mucosal leukocytes, such as αβ T cells and neutrophils (19, 21). Additionally, different γδ T cell subset could has opposite effector functions, for example, Vδ1^+^ γδ T cells that are more prone to immune depression, as a contrast, Vδ2^+^ γδ T cells belongs to cytotoxic subset (23).

According to relevance analyses, we found the Vδ1^+^/Vδ2^+^ ratio and Vδ1^+^PD-1^+^ γδ T cells increased in EP patients, and these two parameters of γδ T cells were associated with pregnancy rate (time) in reproductive EP patients. Specifically, when the Vδ1^+^/Vδ2^+^ ratio is greater than 1.04, the pregnancy time could be delayed for 52 days, from 158 days to 210 days (median pregnancy time, MPT). As for Vδ1^+^PD-1^+^ γδ T cells, however, higher expression of PD-1 molecules in Vδ1^+^ γδ T cells implicates with better pregnancy rate, significantly reduced MPT from 194 days to 130 days, and the threshold valve for Vδ1^+^PD-1^+^ γδ T cells in Vδ1^+^ γδ T cells was set at 15.7 according to statistical median. It’s surprising to note that, in healthy populations without EP, however, the statistical calculation indicated that the pregnancy outcomes associating with Vδ1^+^/Vδ2^+^ ratio and Vδ1^+^PD-1^+^ γδ T cells were opposite comparing to EP. Such opposite γδ T cell phenotype between the EP group and the control group suggested that the balance between Vδ1^+^ γδ T cell and Vδ2^+^ γδ T cell is quite important for pregnancy of women of reproductive age. However, whether functional difference of γδ T cell between two groups led to such opposite phenotype remained to be further verified.

Commonly, majority (>50%) of peripheral γδ T cells carries Vδ2 TCR in human (24), therefore, the Vδ1^+^/Vδ2^+^ ratio is less than 1 in healthy population. According to our unpublished data, however, the Vδ1^+^/Vδ2^+^ ratio is commonly and greatly higher than 1 in cancer patients, implicating with immune suppression effects exerted by Vδ1^+^ γδ T cells. As for suppression aspect of Vδ1^+^ γδ T cell, it possesses regulatory function just like conventional Treg cell. Previous works had also described that this subset can secrete higher amounts of TGF-β than conventional Treg, and can potently suppress naïve and effector T cells responses and meanwhile inhibit maturation of dendritic cells in tumor (24–26). In addition, since γδ T cells at the maternal-fetal during pregnancy are mainly recruited from peripheral blood, it’s logical to hypothesize that circulating γδ T cells would be new markers for postoperative fertility in EP.

Therefore, in healthy population of reproductive age, higher proportion of Vδ1^+^ γδ T cell subset, which can produce immune suppressive effects and thus balance cytotoxic effect of Vδ2^+^ γδ T cell subset, could facilitate fertilization and subsequent embryo implantation. This is consistent with previous observation on circulating Vδ1^+^ γδ T cell in healthy pregnant women (27). In this context, we further found that ≥15.7% of PD-1 expression in Vδ1^+^ γδ T cells adversely affected MPT, and delayed the MPT from 59 days (<15.7 of PD-1 expression) to 113 days. Such results revealed that, in healthy population of reproductive age, PD-1^low^Vδ1^+^ γδ T cells could be potentially developed into a biomarker for predicting the required duration of pregnancy. As a contrast, because of existence of immune disturbance in EP patients, the clinical relevance between pregnancy rate and the Vδ1^+^/Vδ2^+^ ratio as well as PD-1^+^Vδ1^+^ γδ T cell showed to be the opposite when comparing with the healthy populations. We believe that systematical immune cell function suppression contributed crucially to the developed endometrial polyp in women of reproductive age, especially the imbalanced Vδ1^+^ γδ T cell *versus* Vδ2^+^ γδ T cell in peripheral blood. Moreover, compared to healthy controls, higher proportion of Vδ1^+^ γδ T cell and significantly reduced expression of PD-1 in Vδ1^+^ γδ T cell suggested a more potent immune suppression in EP patients, which thus affects pregnancy adversely (lower conception rate and longer interval time). Therefore, among EP patients, those have either lower Vδ1^+^/Vδ2^+^ ratio or higher proportion of PD-1^+^ Vδ1 γδ T cell, which suggests less immune suppressive features of γδ T cells, can lead to better outcome on successive pregnancy. Different from healthy controls, PD-1^high^Vδ1^+^ γδ T cells could be potentially developed into a biomarker for predicting the required duration of post-operative pregnancy for those EP patients who received hysteroscopic polypectomy. It should be mentioned that further large scale of EP patients will be recruited to validate such findings, particularly the clinical relevance of PD-1^+^Vδ1^+^ γδ T cells in predicting fertility in EP population of reproductive age. Notably, we only examined circulating immune cells of EP patients, it’s worthy to unveil whether the infiltrated immune cells including γδ T cells (Vδ1^+^/Vδ2^+^ ratio and PD-1^+^ Vδ1 T) in the endometrium have similar change pattern to the circulating γδ T cell, and the underlying functional features of both circulating and infiltrated γδ T cells are of worthy to be further investigated as well.

In conclusion, the present work suggests the mild imbalance of immune phenotype between Vδ2 and Vδ1 γδ T cells is crucial for successful pregnancy of healthy women of reproductive age. More importantly, the Vδ1/Vδ2 ratio could be a common indicator in both healthy and EP populations of reproductive age to predict the required duration of pregnancy; ≥1.04 of the Vδ1/Vδ2 ratio in healthy women implies shorter MPT, however, <1.04 in EP patients means shorter required time of pregnancy. Furthermore, expression of PD-1 in Vδ1^+^ γδ T cells could be the second prediction biomarker. PD-1^<15.7%^ Vδ1^+^ γδ T cells correlate with shorter pregnancy time (MPT) in healthy populations. As a contrast, PD-1^≥15.7%^ Vδ1^+^ γδ T cells mean shorter MPT in EP patients. Altogether, the Vδ1^+^/Vδ2^+^ ratio and PD-1^+^ Vδ1 T cell could be potentially developed into new immune indicators for predicting the pregnancy rate of EP patients. These indicators could supplement routine clinical examinations including hormone testing and ultrasound imaging, thus provide more comprehensive and precise clinical diagnoses, and eventually benefit EP patients. Finally, the present work revealed hallmarks of peripheral γδ T cell in reproductive females with and without EP, and provided a proof-of-concept for discovering immunological biomarker for predicting fertility clinically.

## Data Availability Statement

The raw data supporting the conclusions of this article will be made available by the authors, without undue reservation.

## Ethics Statement

The studies involving human participants were reviewed and approved by the Shenzhen Baoan Women’s and Children’s Hospital, Jinan University (IRB No: LLSC-2018-08-01). The patients/participants provided their written informed consent to participate in this study.

## Author Contributions

X-HL: project design, sample collection, follow-up, data analysis, and manuscript drafting. M-YL: sample collection, data analysis, and graphs plotting. Y-jL, Z-hL, and Z-nY: data analysis and result discussion. BL: project design and management, data analysis, and manuscript drafting. Y-zW: project design, experimental supervision, data analysis and discussion, and manuscript drafting and revision. All authors contributed to the article and approved the submitted version.

## Funding

This work was supported by Research Foundation of Shenzhen Baoan Women’s and Children’s Hospital, Jinan University to BL (BAFY 2020005).

## Conflict of Interest

The authors declare that the research was conducted in the absence of any commercial or financial relationships that could be construed as a potential conflict of interest.
