# Supplementary material for: Circulating PD1+Vδ1+γδ T Cell Predicts Fertility in Endometrial Polyp Patients of Reproductive-Age
Source: Front Immunol. 2021 Jun 15;12:639221. doi: 10.3389/fimmu.2021.639221 (PMC8239402; doi:10.3389/fimmu.2021.639221)

## Slide 1
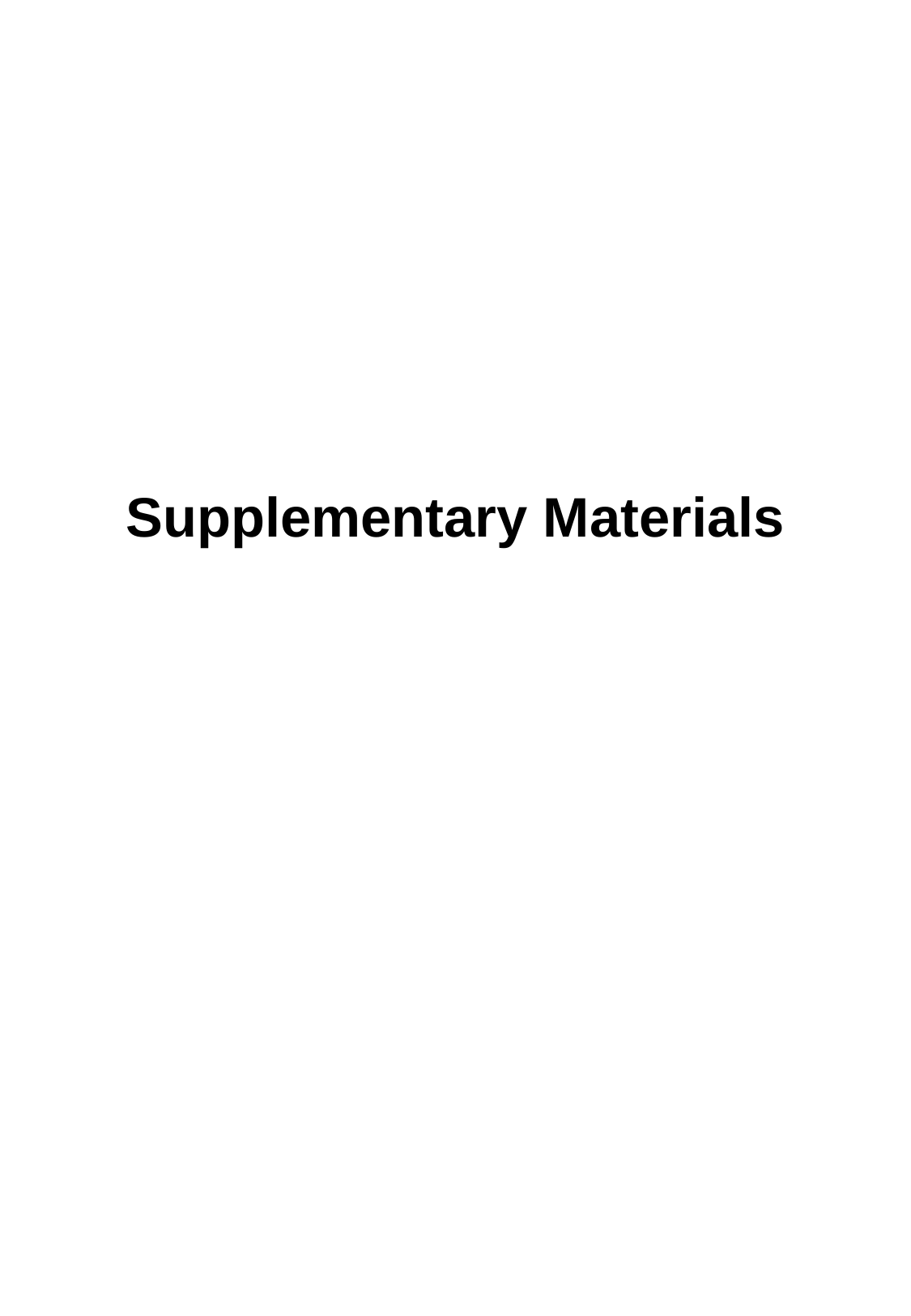

# Supplementary Materials

## Slide 2
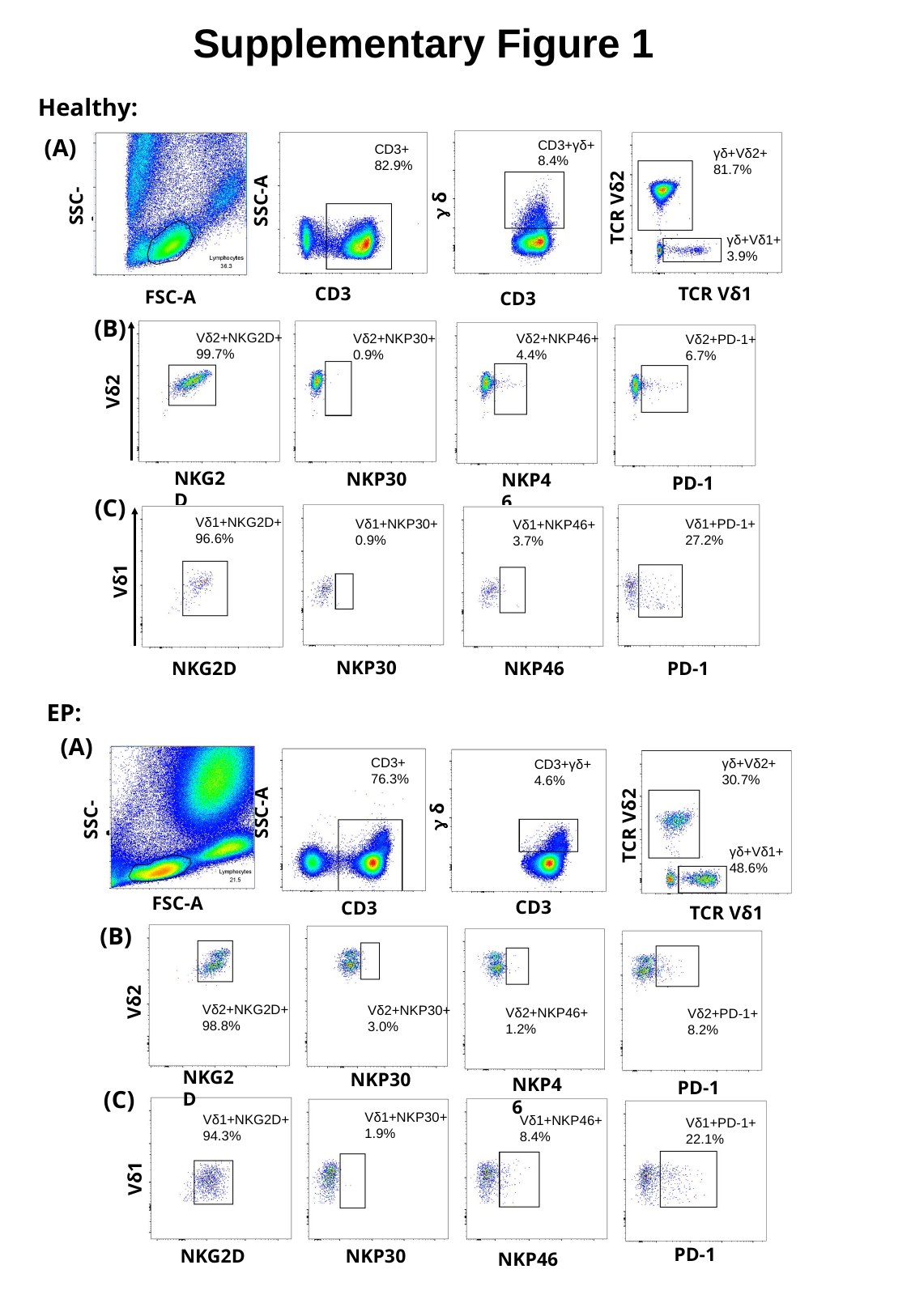

Supplementary Figure 1
Healthy:
(A)
CD3+γδ+
8.4%
CD3+
82.9%
γδ+Vδ2+
81.7%
SSC-A
SSC-A
TCR Vδ2
γ δ
γδ+Vδ1+
3.9%
CD3
TCR Vδ1
FSC-A
CD3
(B)
Vδ2+NKG2D+
99.7%
Vδ2+NKP30+
0.9%
Vδ2+NKP46+
4.4%
Vδ2+PD-1+
6.7%
Vδ2
NKG2D
NKP30
NKP46
PD-1
(C)
Vδ1+NKG2D+
96.6%
Vδ1+PD-1+
27.2%
Vδ1+NKP30+
0.9%
Vδ1+NKP46+
3.7%
Vδ1
NKP30
NKG2D
NKP46
PD-1
EP:
(A)
CD3+
76.3%
γδ+Vδ2+
30.7%
CD3+γδ+
4.6%
SSC-A
SSC-A
γ δ
TCR Vδ2
γδ+Vδ1+
48.6%
FSC-A
CD3
CD3
TCR Vδ1
(B)
Vδ2
Vδ2+NKG2D+
98.8%
Vδ2+NKP30+
3.0%
Vδ2+NKP46+
1.2%
Vδ2+PD-1+
8.2%
NKG2D
NKP30
NKP46
PD-1
(C)
Vδ1+NKP30+
1.9%
Vδ1+NKG2D+
94.3%
Vδ1+NKP46+
8.4%
Vδ1+PD-1+
22.1%
Vδ1
PD-1
NKG2D
NKP30
NKP46

## Slide 3
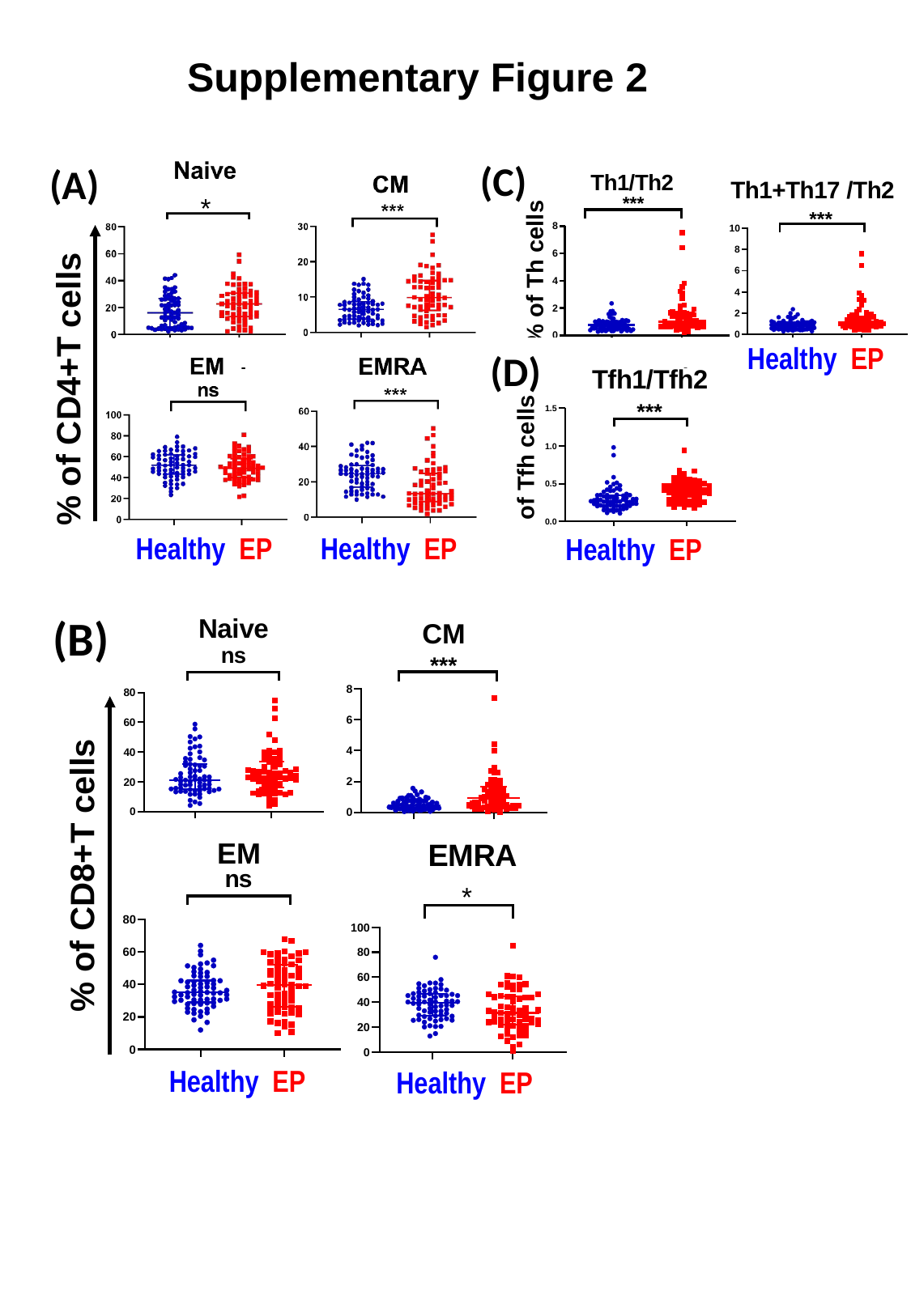

Supplementary Figure 2
(C)
(A)
*
% of Th cells
Healthy EP
(D)
% of CD4+T cells
% of Tfh cells
Healthy EP
Healthy EP
Healthy EP
(B)
% of CD8+T cells
*
Healthy EP
Healthy EP

## Slide 4
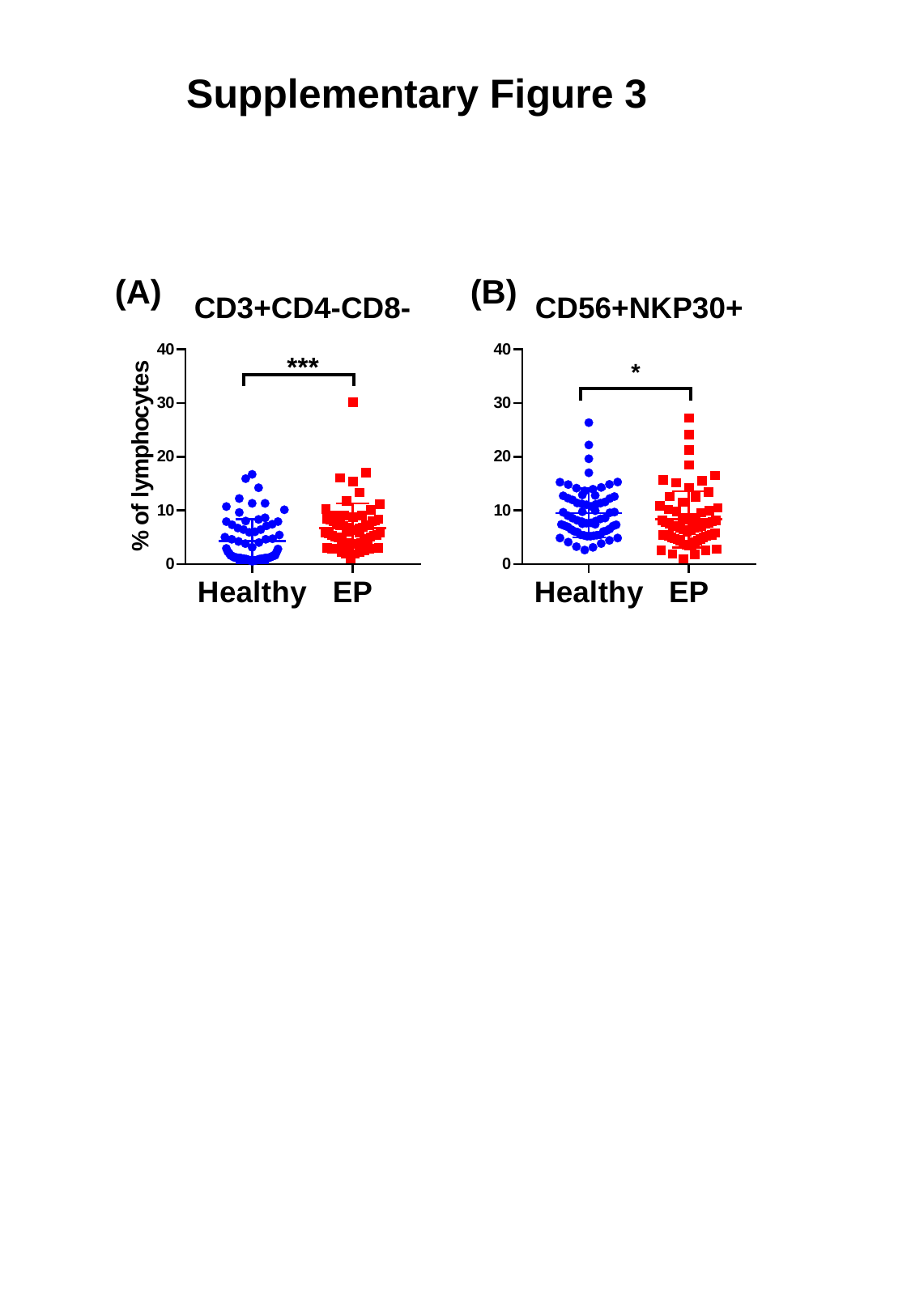

Supplementary Figure 3
(A)
(B)

## Slide 5
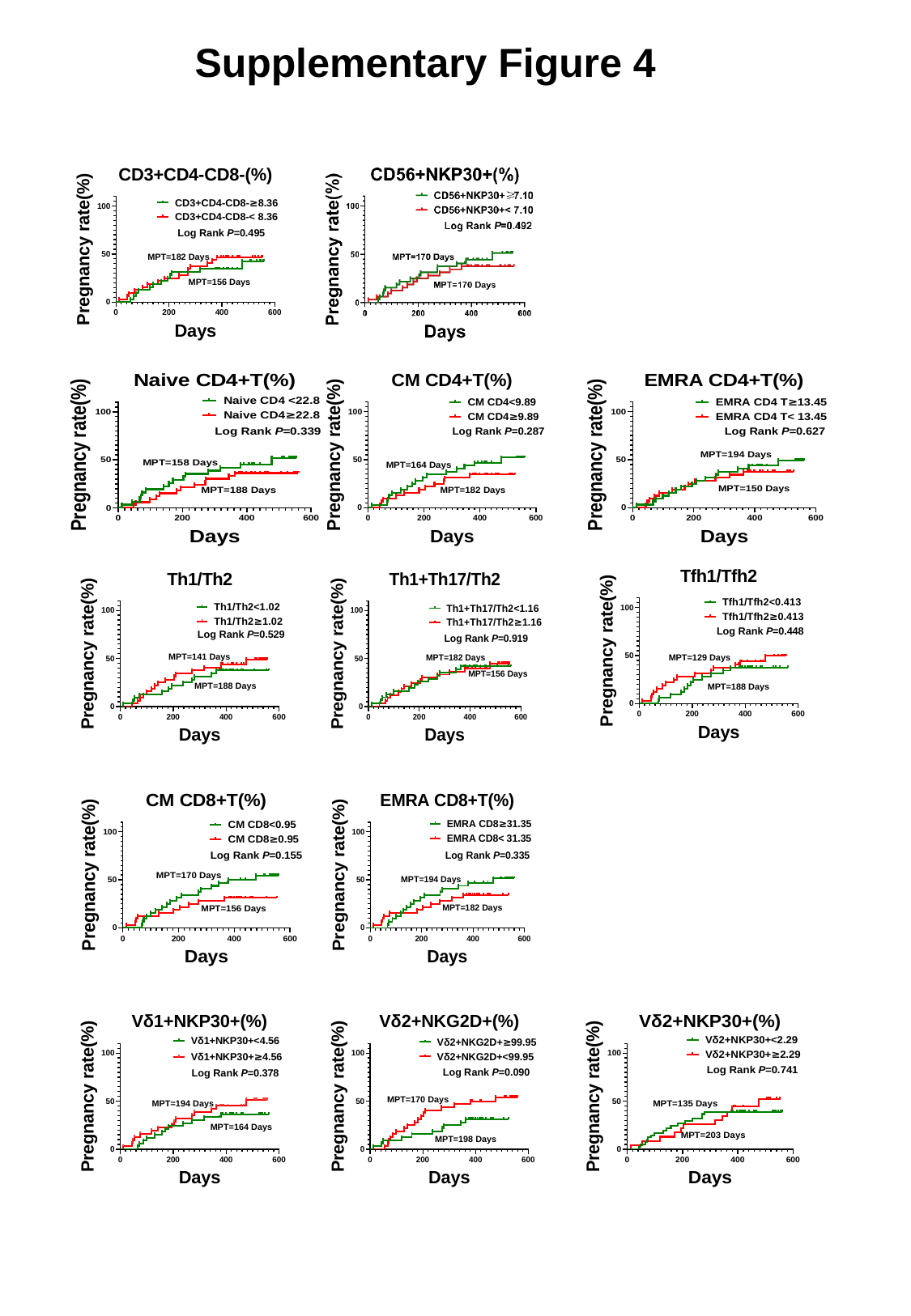

Supplementary Figure 4

## Slide 6
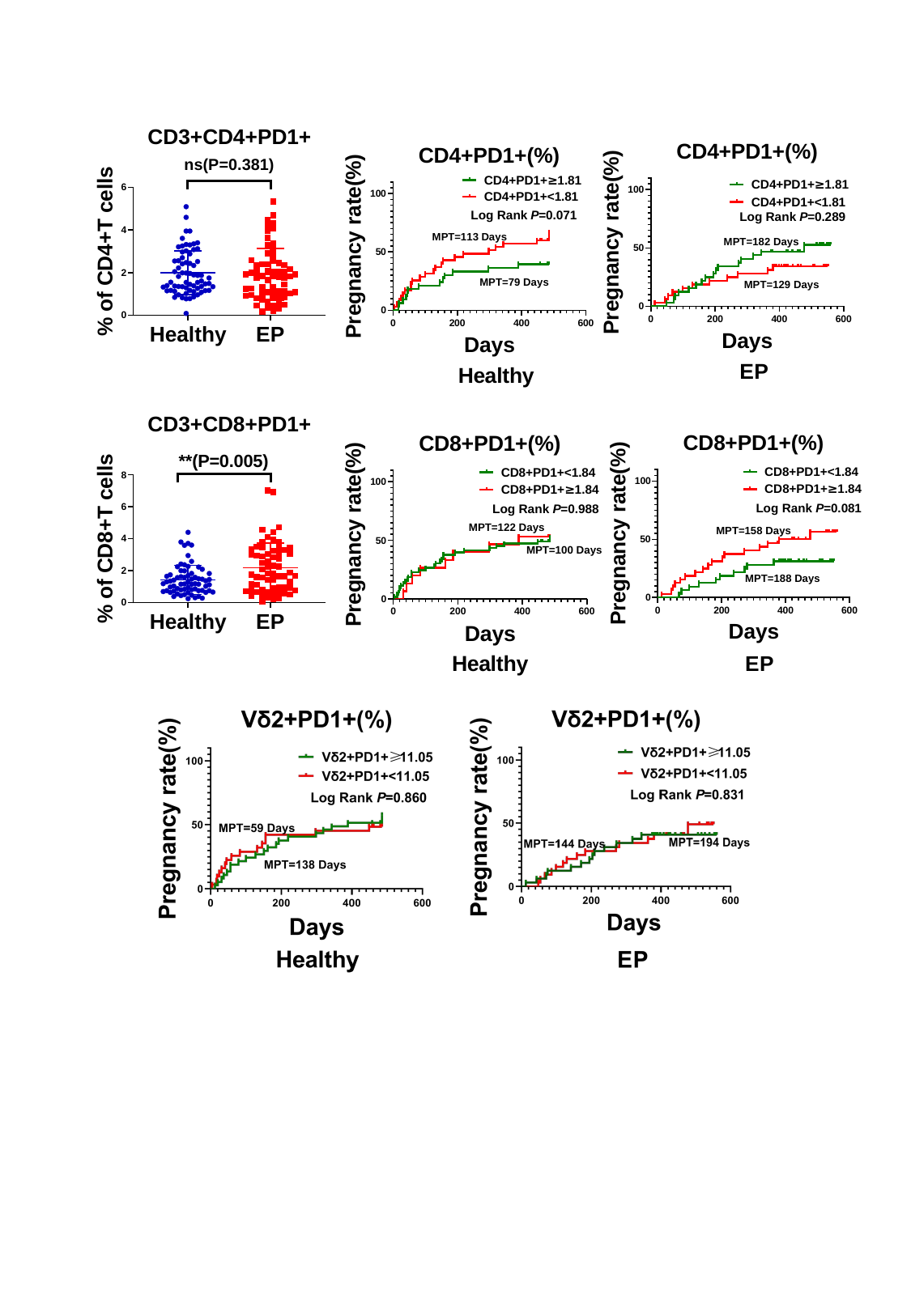

Supplement: Supplementary file 1 [file Presentation_1.pptx]
